# Long term results of total hip arthroplasty with cemented and cementless tapered femoral component

**DOI:** 10.1051/sicotj/2023014

**Published:** 2023-06-05

**Authors:** Shai Factor, Tal Frenkel Rutenberg, Simon Garceau, Aviram Gold, Samuel Morgan, Nimrod Snir, Yaniv Warschawski

**Affiliations:** 1 Division of Orthopedic Surgery, Tel Aviv Medical Center Tel Aviv 6423906 Israel; Affiliated to Sackler Faculty of Medicine, Tel Aviv University Tel Aviv Israel; 2 Rabin Medical Center, Division of Orthopaedics Petach Tikva Israel; 3 Mount Sinai Hospital, Division of Orthopaedics – Joseph and Wolf Lebovic Health Complex Toronto ON Canada

**Keywords:** Taperloc, Total hip arthroplasties, Surgery, survival, long-term outcomes

## Abstract

*Background*: Excellent midterm results for total hip arthroplasties (THA) with cementless, tapered porous Taperloc^®^ femoral stems have been reported. Reports regarding such cemented stems, however, are lacking. *Objectives*: To evaluate the long-term outcomes of both cemented and cementless THAs with the Taperloc femoral component. *Methods*: The medical records of 71 patients (76 hips), operated on between January 1991 and December 2003, who had a minimum follow-up of 10 years were available for analysis. Functional analysis was performed with the Harris hip score (HHS) questionnaire and the numerical analogue scale (NAS). Radiographic analysis was performed for subsidence, radiolucent lines and osteolysis. *Results*: The cohort was comprised of 47 female and 24 male patients, with a mean age of 59.7 ± 12.4 years. The mean follow-up was 17.8 ± 4.4 years. 52.6% of THAs analyzed were cementless and 47.4% were cemented. Post-operative radiographs were available for 57 surgeries. Subsidence, hypertrophic ossification, radiolucent lines and osteolysis were noted in 4 (7%), 2 (2.6%), 14 (18.4%) and 11 (14.5%) hips respectively. The average HHS score at a mean follow-up of 20.1 ± 3.9 years was 62.1 (±27.7) and the NAS score was 4.6 (±3.6). During the study period, five revision surgeries were performed due to stem-related problems, one of which was for aseptic loosening. *Conclusions*: Our long-term experience with the Taperloc stem, both cemented and cementless, demonstrates good outcomes, with low rates of failure. This makes this prosthesis an attractive option for THAs. Level of Evidence: IV

## Introduction

Total hip arthroplasty (THA) is a surgical procedure that has improved the quality of life for millions of individuals worldwide [1–3]. Since its introduction by Wiles in 1938, numerous designs and materials have been used. Contemporary implants have achieved excellent clinical outcomes and longevity using both cemented and uncemented prostheses [4–6]. The Taperloc^®^ femoral component, available in both cemented and uncemented versions, was developed in collaboration with Dr. Richard H. Rothman and is marketed by Biomet (Zimmer Biomet, Warsaw, IN) [7]. This non-collared implant is composed of Ti6Al4V titanium alloy. In its uncemented form, the stem’s rectangular shape is designed to achieve mediolateral fixation within the metaphysis of the proximal femur. Additionally, flattening in the anteroposterior direction increases rotational stability. The proximal third of the implant is coated with titanium alloy applied with a pressure plasma spray technique and is designed to promote initial stability and bone fixation. The distal part of the stem has a smooth polish finish. The cemented implant, which is non-collared, is made of cobalt chrome and is polished. It has a tapered shape for optimal stability and utilizes the same conventional broaching system as the cementless implant [8, 9].

Since its introduction, several authors have reported on the outcomes of the Taperloc cementless implant, with increasing duration of follow-up. Most have reported excellent survival rates and hip scores for both young patient and elderly patients [8–13]. Labek et al. [7] performed a comprehensive literature review on the outcome of the cementless Taperloc stem demonstrating good performance in regards to revision rates. Their study noted a limitation was that a large proportion of their results were published by a single institution, limiting the reproducibility by other surgeons. The authors suggested data from other registries will add important information to knowledge on the performance of this implant. Moreover, while much of the current literature has described outcomes of cementless Taperloc implants, there is currently a paucity of high-quality studies reporting outcomes on the cemented Taperloc implant. Both available cemented studies describe their short to mid-term results using Boneloc^®^ (Biomet, Bridgend, South Wales, UK) cement which was withdrawn from the market in 1995 due to early loosening and failure of the femoral stem [14, 15]. The purpose of this manuscript is to report the results of a long-term clinical study (average follow-up of 18 years, range 10.6–26.6 years) describing the outcomes of the Taperloc stem in both its cementless and cemented forms from an institutional registry, with a specific focus on implant survival.

## Methods

### Demographics

Following approval by our institutional review board, we reviewed the records of patients treated with primary THA using a Taperloc stem between January 1991 and December 2003, at our tertiary hospital.

The surgical approach used for all procedures was the posterolateral approach. A total of four surgeons performed the primary THA surgeries during the study period. Patient demographics that were collected included gender, age, indication for surgery, follow-up period and mortality. Additionally, we collected data regarding implant characteristics including stem size, neck size, number of acetabular screws, head size, head material and cup material. Additionally, information was collected from the patients’ files regarding revision surgeries, including the reasons for undergoing revision, such as periprosthetic fracture, infection, aseptic loosening, and implant subsidence.

The records of 324 consecutive patients (338 hips) were reviewed. 253 patients (262 hips) did not meet the minimum follow-up duration of 10 years. Eighty patients had died prior to the 10-year follow-up, and an additional 91 patients (96 hips) had passed away prior to data collection. Therefore, a total of 71 patients (76 hips) consisting of 38 (40 hips) cementless THAs and 36 (36 hips) cemented THAs were available for analysis, all cups used were uncemented. The resultant cohort was comprised of 47 female and 24 male patients. The mean age was 59.7 ± 12.4 years (range, 25.7–84.0 years). The mean follow-up was 17.8 ± 4.4 years (range, 10.6–26.6 years). The most common indication for THA was osteoarthritis (31 hips) followed by avascular necrosis of the femoral head (15 hips), developmental dysplasia of the hip (11 hips), failed THA requiring revision surgery (9), rheumatoid arthritis (5), and others (5 hips). Demographic and Implant characteristics are presented in Tables 1 and 2.

**Table 1:** Patient demographics.

	Cementless group (*n* = 38)	Cemented group (*n* = 36)	*p*-Value
Age (years)	58.6 ± 13.1	61.0 ± 11.4	0.21
Follow-up (years)	17.8 ± 4.4	17.8 ± 4.4	0.98
Indication for THA, *n* (%)			
Osteoarthritis	13 (32.5)	18 (50.0)	0.16
Avascular necrosis	8 (20.0)	7 (19.4)	0.94
Developmental dysplasia	8 (20.0)	3 (8.3)	0.17
Failed THA	6 (15.0)	3 (8.3)	0.51
Rheumatoid arthritis	1 (2.5)	4 (11.1)	0.28
Others	4 (10.0)	1 (2.8)	0.29

Data presented as mean ± SD.

**Table 2:** Implant characteristics.

Stem size *n*, (%)	5	1 (1.3)
	7.5	18 (23.7)
	10	32 (42.1)
	12.5	18 (23.7)
	15+	4 (5.3)
	NA	3 (3.9)
Neck size *n* (%)	Short	6 (7.9)
	Normal	60 (78.9)
	Long	7 (9.2)
	NA	3 (3.9)
Acetabular screw *n* (%)		14 (18.4)
Head size *n* (%)	22	4 (5.3)
	28	63 (82.9)
	32	4 (5.3)
	NA	5 (6.6)
Head material *n* (%)	Ceramic	9 (11.8)
	Cobalt chrome	59 (77.6)
	Metal	2 (2.6)
	NA	6 (7.9)
Cup material	Porous coated	60 (78.9)
	Titanium	5 (6.6)
	ARCOM UHMWPE	2 (2.6)
	NA	11 (14.5)

**Table 3:** Imaging analysis.

Gruen zone	Radiolucent line, *n* (%)			Osteolysis, *n* (%)		
	All (*n* = 57)	Cementless (*n* = 29)	Cemented (*n* = 28)	All (*n* = 57)	Cementless (*n* = 29)	Cemented (*n* = 28)
1	8 (14.0)	2 (6.9)	6 (21.4)	9 (15.8)	3 (10.3)	6 (21.4)
2	7 (12.3)	0	7 (25.0)	5 (8.8)	0	5 (17.9)
3	3 (5.3)	0	3 (10.7)	4 (7.0)	0	4 (14.3)
4	3 (5.3)	0	3 (10.7)	4 (7.0)	0	4 (14.3)
5	3 (5.3)	0	3 (10.7)	5 (8.8)	1 (3.4)	4 (14.3)
6	6 (10.5)	0	6 (21.4)	5 (8.8)	1 (3.4)	4 (14.3)
7	10 (17.5)	2 (6.9)	8 (28.6)	7 (12.3)	2 (6.9)	5 (17.9)

### Radiographic evaluation

Anteroposterior and lateral X-rays of the operated hip were reviewed by the senior surgeon. Changes around the femoral and acetabular components were documented, including the presence of radiolucent lines, endosteal new bone formation near the prosthesis (spot welds), and subsidence. To evaluate subsidence, the vertical distance between the top of the greater trochanter and the top of the stem was measured. Positive stem subsidence was defined as a vertical distance greater than 5 mm [16]. Implant survival analysis was calculated separately for the stem and all other components (head, liner, etc.)

### Functional analysis

Preoperative functional evaluation was performed using the modified Harris Hip Score (HHS) [17] and the Numeral Analogue Scale (NAS).

### Statistical analysis

Continuous variables are presented as means and standard deviations. Categorical variables were presented as absolute and relative frequencies. Comparisons between categorical variables were performed using the Fisher exact test and between continuous variables with the Two-tailed Student’s t-test. Kaplan–Meier survival curves were used to assess implant survival. Statistical significance was defined as *p* < 0.05. Survival curves were created using the Survminer package for R.

## Results

### Radiographic evaluation

Post-operative radiographs were available for 57 surgeries, 29 (72.5%) in the uncemented group and 28 (77.8%) in the cemented group. The average time lapse between surgery and imaging was 15.4 ± 4.4 years. Subsidence of more than 2 mm was noted in 4 patients (7%), one of them cementless (3.4%) and the remainder cemented (10.7%). Hypertrophic ossification was evident in two patients (2.6%), both in the cementless group. Radiolucent lines were evident in 14 (24.6%) patients, with a significantly higher prevalence in the cemented group (12 patients, 42.8%) compared to the cementless group (2 patients, 6.9%) (*p* = 0.026) Osteolysis, defined as a circular or oval area of evident bone loss, was seen in 11 (19.3%) patients, 4 (13.8%) from the cementless group and 7 (25.0%) from the cemented group. The location of radiolucent lines and osteolysis relative to the in-situ implants is demonstrated in Table 2.

### Functional assessment

Among the 53 patients (58 hips) who remained alive at the end of the study, 42 (79.2%) were available for completion of the Harris Hip Score (HHS) and the NAS questionnaires. The average HHS and NAS scores at an average of 20.1 ± 3.9 years (14.1–26.6) post-operatively were 62.1 (±27.7) and 4.6 (±3.6), respectively. Notably, a dozen patients (22.6%) who completed the outcome scores had previous revision surgery. When comparing the scores of the cementless and cemented hip patients, no significant difference was noted for both outcomes, the *p*-value of 0.597 for the HHS (average score for both groups respectively was 64.2 ± 25.8 and 59.4 ± 30.6) and *p*-value of 0.884 for the NAS score (average score for both groups respectively was 4.7 ± 3.6 and 4.5 ± 3.6).

### Implant survival

**Figure 1:**
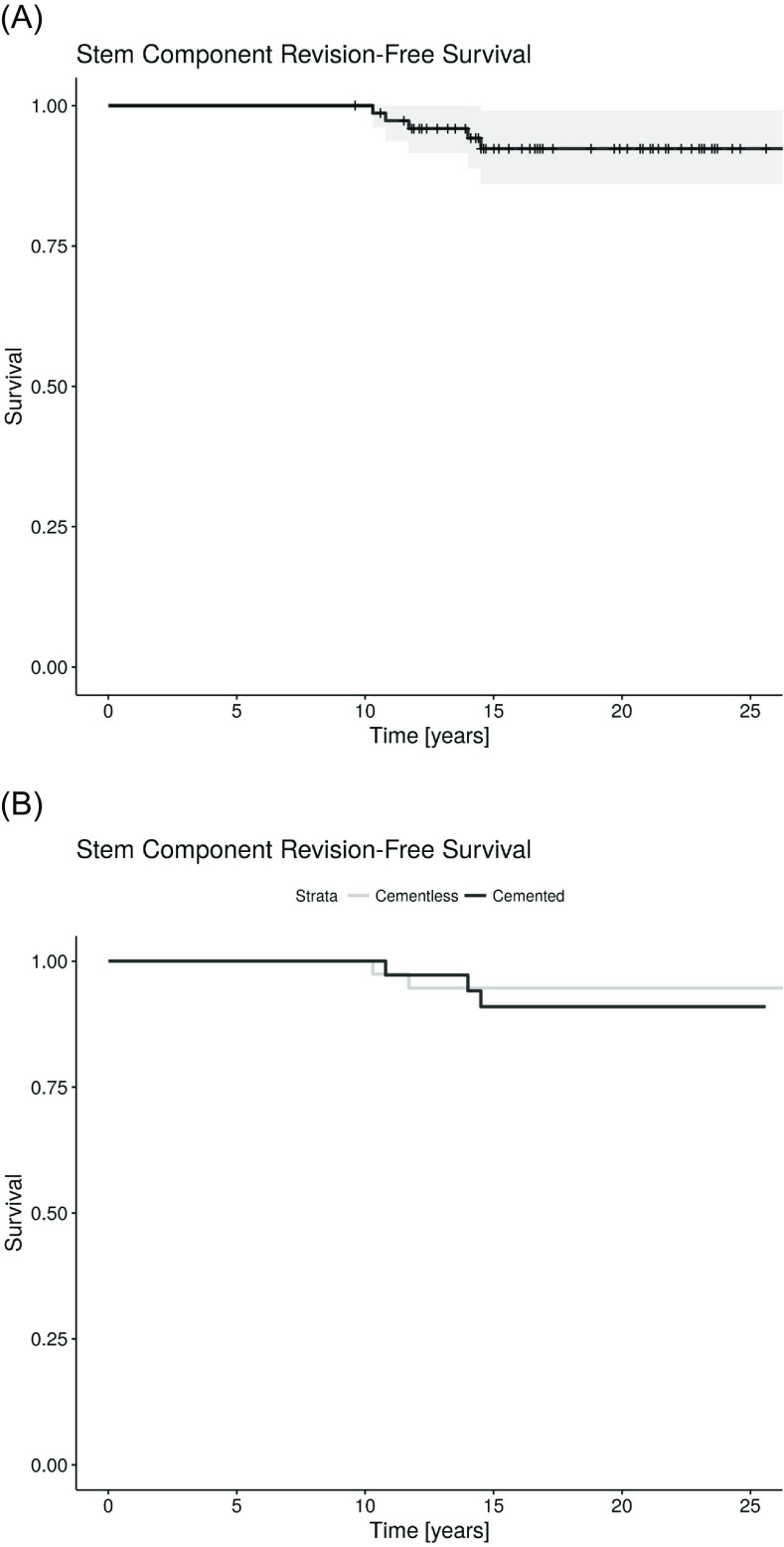
Femoral component survival. A) During an average follow-up of 17.8 years, 5 stems were revised. B) The first two revisions were for cementless stems and the latter three for cemented implants.

**Figure 2:**
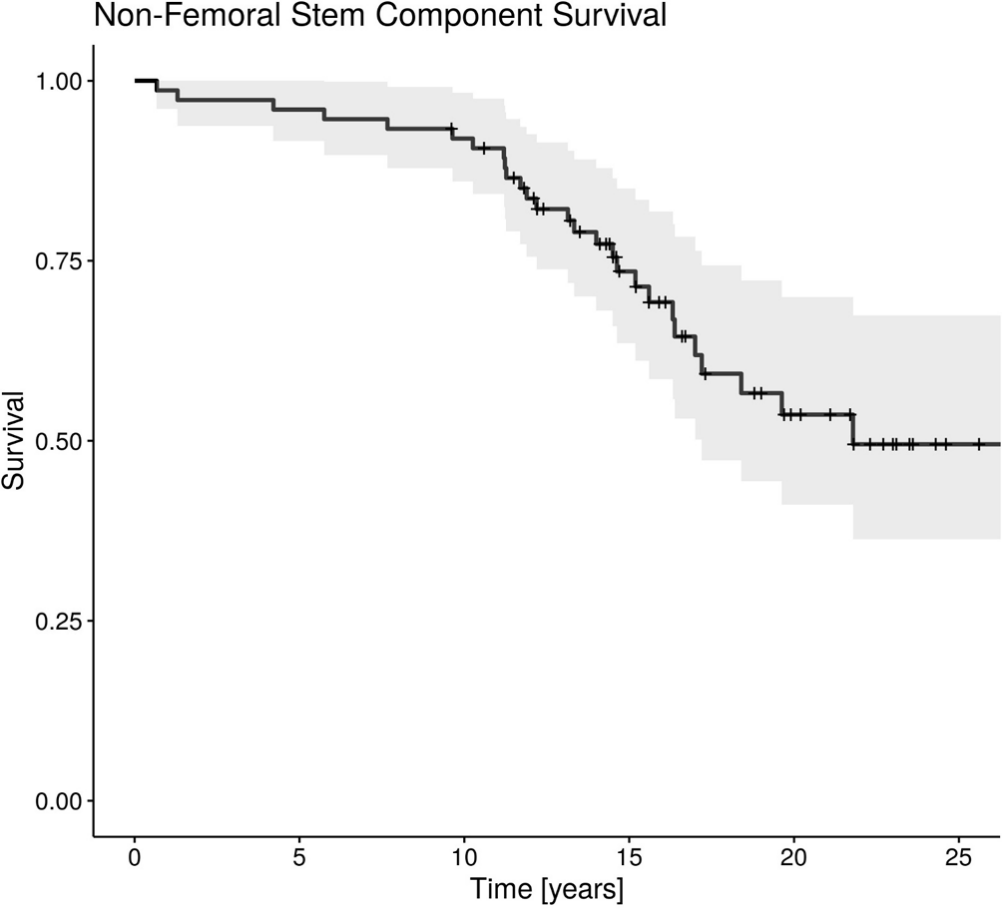
Non-femoral component survival. During an average follow-up of 17.8 years, 20 revisions for non-stem-related problems were performed, and 8 additional patients with non-stem-related complications did not undergo revision.

During the study period, 5 revision surgeries (6.6% failures) were performed due to stem-related problems; 1 infection, 1 subsidence, 1 periprosthetic fracture, 1 aseptic loosening, and in one patient the cause of failure was unavailable as the revision was performed at an outside centre. The first two revisions were for cementless stems (5.0%) and the latter three for cemented implants (8.3%). The average time to stem failure was 12.3 ± 1.9 years (range 10.3–14.5 years). Twenty revision surgeries (26.3% failures) were performed due to non-stem related problems, with the leading cause being polyethylene wear. The average time to revision was 11.3 ± 5.6 years (range 0.7–21.8 years). Notably, seven additional patients were diagnosed with polyethylene wear and an additional patient with osteolysis in the acetabular region during follow-up. These patients, however, did not undergo revision surgery. The inclusion of these patients in the analysis increases the failure rate of the non-stem components to 36.8% (Figures 1 and 2).

**Figure 3:**
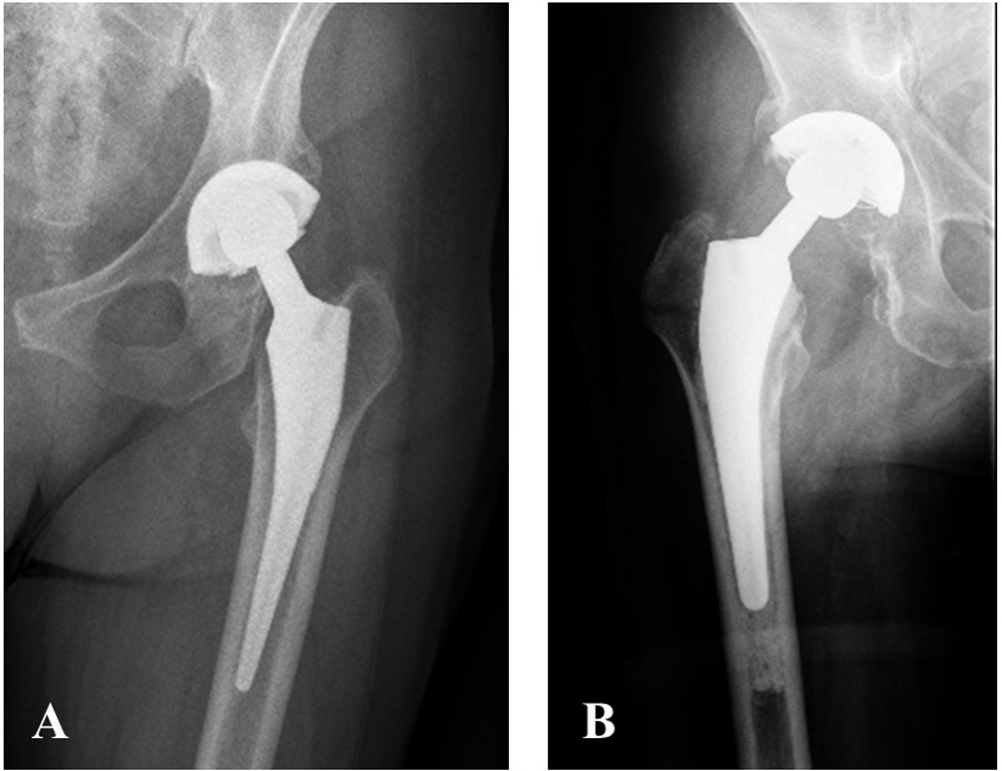
A) A 13-year follow-up radiograph of the left hip of a female patient with a cementless Taperloc stem. B) A 12-year follow-up radiograph of the right hip of a male patient with a cemented Taperloc stem.

## Discussion

Excellent midterm results for total hip arthroplasties (THA) with cementless, tapered porous Taperloc^®^ femoral stems have been reported. Reports regarding such cemented stems, however, are lacking. The purpose of the current was to evaluate the long-term outcomes of both cemented and cementless THAs with the Taperloc femoral component. We found good long-term outcomes for the Taperloc femoral component, with survival rates of 93.4% at an average of 17.8 years postoperatively. This was true both for cementless and cemented stems.

While our results demonstrate high survival rates, they are still lower than those reported by others. Labek et al. [7], reported that survival rates reported by the developers appeared superior to those reported by other surgeons. Indeed, Purtill et al. [11] and Parvizi et al. [12] have reported lower revision rates of 0.6% and 0.9% respectively for the cementless femoral component, yet their average follow-up duration was shorter, 11 years. On the other hand, McLaughlin and Lee [18] reported a higher revision rate of 9% (for any cause) when the follow-up period was extended to 20 years. Still, when revision rates due to aseptic loosening are analyzed, our rates are similar to others [9, 10, 19–21], as this was the reason for only a single surgery (1.3%). Regarding the non-stem components, survival was lower at 63.2% with liner wear being the major cause for failure and revision.

While we did not find any data regarding the long-term survival of the Taperloc cemented THA, or a comparison of its performance with the Taperloc cementless stem, Corten et al. [22] presented a long-term follow-up for another tapered stem. The authors assessed both cemented (*n* = 52) and cementless (*n* = 41) Mallory-Head total hip replacements. In their cohort, they found that the cementless stems achieved superior survival. Similarly, results for the Mallory-Head total hip replacements were presented by Laupacis et al. [23] at a mean follow-up of 6.3 years. In their work, the major cause for cemented stem revision was loosening, while only one cementless stem was revised. The revision of the cementless stem was due to a periprosthetic fracture. In our cohort, stem revision rates were similar between groups, 95% for cementless stems and 91.7% for cemented stems (*p*-value = 0.663). The follow-up duration was longer for the cemented group (16.4 ± 4.2 vs. 19.4 ± 4.4 years for both groups, *p*-value = 0.002).

HHS scores in our cohort were lower than those reported by others. McLaughlin et al. [8] reported an average score of 92 at a 25-year follow-up. The cohort analyzed in this study, however, included younger patients (<50 years old) and patients who underwent revision were excluded from the analysis. Others [11–13] who presented superior scores of 82–92 had a shorter follow-up of 5–11 years. A possible explanation for our poorer results may be the high rate of polyethylene wear observed. Earlier surgeries in our patient cohort were not all performed using highly linked polyethylene until this became routine practice at our centre. This may have led to increased hip pain and non-stem-related revision rates.

The current study has several limitations. Due to the long follow-up period, a significant proportion of patients were lost to follow-up, either due to death or failure to attend routine follow-up appointments after the first post-operative years. This may have resulted in biased survival results. Additionally, not all patients with sufficient follow-up were available for functional assessment, which may have impacted our analysis of postoperative outcomes. Another limitation of this study was the lack of recently available radiographs for all patients, which could have helped to provide more accurate and up-to-date information. Moreover, our study had only one observer, which may have introduced some degree of variability and reduced the generalizability of our findings to other settings. Nonetheless, we took several measures to minimize potential bias and error, such as selecting a senior and experienced surgeon as the observer and using standardized methods for measurement. Furthermore, we did not perform an analysis to explore the association between osteolysis and the type of polyethylene liner and femur morphology. Finally, the lack of a control prosthesis group limits the ability to make direct comparisons between the Taperloc implant and other types of implants. Despite these limitations, we believe outcomes from our institutional registry add notable value to orthopaedic knowledge regarding the performance of these implants, in particular for the cemented Taperloc implant which has not been extensively studied in the current body of literature.

## Conclusions

Our long-term experience with the Taperloc stem, both cemented and cementless, demonstrates good outcomes and survivorship making it an attractive choice for THAs.
